# Editorial: Gamification, social skills and mental health promotion

**DOI:** 10.3389/fpsyt.2024.1441344

**Published:** 2024-07-26

**Authors:** Victor Otani, Ricardo Uchida, João Vissoci

**Affiliations:** ^1^ Faculty of Medical Sciences, Santa Casa of Sao Paulo, São Paulo, Brazil; ^2^ Department of Emergency Medicine, Duke University, Durham, NC, United States

**Keywords:** social skills, gamification, RPG, mental health promotion, serious games

## Rethinking traditional treatments

Despite significant advancements in pharmacological treatments for mental health conditions, a substantial number of individuals continue to struggle to achieve full recovery. This ongoing challenge underscores the considerable gaps and limitations in our current herapeutic approaches (Bergmann et al., Djamnezhad et al.). Traditional pharmacological treatments play a crucial role in managing and stabilizing symptoms of mental health conditions (Bergmann et al.). However, they often fall short in promoting overall mental health and well-being. While these treatments effectively address symptoms, they frequently fail to foster the development of essential social skills, resilience, and healthy lifestyle habits necessary for long-term mental health. This shortfall arises because these treatments do not consider or address these aspects whatsoever. By neglecting these crucial areas, treatments do not adequately tackle the underlying factors contributing to mental health issues, such as social isolation, lack of coping mechanisms, and unhealthy lifestyle choices. Consequently, many individuals find that although their immediate symptoms may be managed, their overall quality of life and ability to cope with life’s challenges do not significantly improve.

## The role of social skills, socialization, and leisure

Social skills, socialization, and leisure activities are fundamental to mental health. Numerous studies have demonstrated that strong social skills and frequent social interactions are associated with better mental health outcomes, including lower rates of depression and anxiety (Djamnezhad et al.). Additionally, these elements play a crucial role in maintaining a healthy work-life balance, which is essential for overall well-being. However, promoting lifestyle changes that incorporate these elements can be challenging, especially for individuals with mental health issues who may find it difficult to engage in social activities or adopt new, healthy habits (Bergmann et al., Djamnezhad et al.).

To effectively promote these changes, it is crucial to connect emotions with learning and behavior. This is where gamification comes into play and becomes a truly invaluable resource. By integrating game elements such as points, levels, and rewards into educational and therapeutic activities, gamification makes these activities more engaging and enjoyable (Djamnezhad et al.). Even though games are frequently perceived as unproductive or of little practical benefit, their integration into therapeutic settings can yield significant mental health benefits. Gamification, by incorporating elements such as points, levels, and rewards into educational and therapeutic activities, transforms mundane or challenging tasks into engaging and rewarding experiences. Not only does this approach enhance motivation for individuals to adopt healthier lifestyles, but it also contributes to improved mental health outcomes. Furthermore, the sustained engagement facilitated by gamification promotes the maintenance of emotional balance and overall mental health, thereby reducing the likelihood of crises or relapses (Bergmann et al., Djamnezhad et al.).

## Integrating gamification with mental health promotion

The research articles featured in this Research Topic of Frontiers on “Gamification, Social Skills, and Mental Health Promotion” collectively underscore the significant, yet often underappreciated, potential of gamification in enhancing mental health outcomes. The findings illustrate how gamified interventions can effectively mitigate symptoms of mental health disorders, enhance cognitive functions, and promote positive behavioral changes.

Evidence from various studies indicates that video game-based interventions can significantly reduce depressive symptoms, enhance training motivation, and improve visuo-spatial memory in patients with Major Depressive Disorder (MDD) (Bergmann et al.). These findings suggest that gamification can serve as a valuable adjunct to traditional therapeutic approaches by engaging patients in a stimulating and enjoyable manner, thereby enhancing the overall effectiveness of mental health treatments.

In educational settings, gamification has proven to be significantly effective in reducing conduct problems, enhancing classroom climate, and increasing teacher efficacy through initiatives such as the Good Behavior Game (GBG) (Djamnezhad et al.). These interventions underscore the expansive applicability of gamification beyond clinical settings, demonstrating its valuable role in preventive and developmental contexts.

Other studies in this topic the cognitive and social benefits associated with gamified activities. Research demonstrates that video games can enhance attention, memory, and problem-solving abilities, all of which are crucial for managing and alleviating symptoms of mental health disorders (Bergmann et al., Djamnezhad et al.). Furthermore, the interactive and immersive qualities of these games foster the development of essential social skills and the formation of supportive networks, thereby contributing to overall mental well-being.

Additionally, the potential of analog games, particularly tabletop role-playing games (ttRPGs), warrants attention. These games offer rich, immersive experiences that enhance social interaction, empathy, and collaborative problem-solving, thus supporting mental health promotion.

The consistency of findings across multiple studies presents a compelling argument for the integration of gamification as a mainstream strategy in mental health care. It is imperative for researchers, clinicians, and policymakers to recognize and harness the transformative potential of gamification to improve mental health outcomes.

## A call for serious scientific inquiry into gamification

It is long overdue for the scientific community to earnestly embrace gamification, particularly simulation approaches like role-playing games (RPGs). The compelling evidence presented in this Research Topic highlights the transformative potential of gamification in promoting mental health. By fostering engagement, motivation, and positive behavioral changes, gamification can significantly complement traditional treatments and introduce innovative pathways for mental health care. The time to integrate games into scientific discourse is now, bringing their benefits fully into the realm of evidence-based practice.

Using gamification in mental health interventions represents a promising frontier that deserves rigorous research and policy support. As we continue to explore its applications and benefits, it is crucial to ensure that gamification is integrated thoughtfully and ethically into mental health strategies. This integration will require collaboration among researchers, clinicians, educators, and policymakers to develop evidence-based practices and guidelines for the effective use of gamification in mental health promotion.

Moreover, addressing potential challenges and ethical considerations associated with gamification is essential. These include ensuring that game content is appropriate and does not promote harmful behaviors, addressing concerns about screen time and addiction, and ensuring that gamified interventions are accessible to diverse populations. By tackling these issues, we can maximize the positive impacts of gamification while minimizing potential risks.

The future of mental health care may well depend on the ability to harness the power of gamification to promote lasting well-being and resilience. By taking gamification seriously, new possibilities for enhancing mental health and improving the quality of life for individuals and communities can be unlocked. This approach has the potential to revolutionize mental health care by making interventions more engaging, effective, and accessible to a broader audience.

## Conclusion

Gamification represents a transformative approach to mental health promotion, offering innovative solutions to enhance cognitive and emotional well-being. The contributions in this Research Topic highlight the diverse applications and significant benefits of gamification. It is crucial for the scientific community to embrace and advance this field, ensuring that gamification is harnessed effectively to promote mental health on a broader scale.

By recognizing the potential of gamification and supporting its integration into mental health strategies, more engaging, effective, and sustainable interventions can be created. Gamification has the potential to make therapeutic activities more enjoyable and interactive, thereby increasing patient engagement and adherence to treatment protocols. This approach can also help in building essential skills such as resilience, social interaction, and stress management, which are critical for long-term mental health.

Ultimately, the future of mental health care may hinge on the ability to innovate and embrace new approaches such as gamification to enhance the well-being and resilience of individuals and communities. The studies presented in this Research Topic are merely the beginning; continued research and collaboration will be vital to realizing the full potential of gamification in mental health promotion. By expanding the understanding and application of gamified interventions, it may be possible to develop more personalized and adaptive mental health care solutions that cater to the unique needs of each individual, ultimately leading to improved mental health outcomes on a global scale (Otani et al.).

## Author contributions

VO: Conceptualization, Writing – original draft, Writing – review & editing. RU: Writing – original draft, Writing – review & editing. JV: Writing – original draft, Writing – review & editing.

